# New Drugs, Appliances, and Things Medical

**Published:** 1892-10-22

**Authors:** 


### NEW DRUGS, APPLIANCES, AND THINGS MEDICAL.

[All preparations, appliances, novelties, etc., of which a notice is desired, should be sent for the Editor, to care of The Manager, 140, Strand, London, W.C.

### THE "CARBOLINE" DISINFECTOR.

Henry Ellison, Cleckheaton, has favoured us with a drawing of the above. We consider that it is an admirable invention. It consists of a "tip-over" lever and float and a small receptacle for attachment to the flush-out cistern to be found in all modern waterclosets. It appears to us to have several advantages, (1) simplicity; it cannot get out of order; (2) economy and exactitude; it always puts the same quantity of "carboline" into the cistern each time the watercloset is used; (3) ease of fixing; it just hangs on the cistern and it can be seen at a glance when in requires refilling. Also, the disinfector does not require to be taken down to refilled, there are no taps, plugs, or nuts to undo or valves to clean. We consider that it is one of the most efficacious and certainly the simplest disinfectors we have met with, and combined with the use of "carboline," which we noticed favourably some time back, ought to render every closet sweet and wholesome.

